# Saving Implants BMP-2 Application in Revision Total Hip Surgery

**Published:** 2006-06

**Authors:** M. Jäger, R. Emami, F. Thorey, R. Krauspe

**Affiliations:** *Department of Orthopedics, Heinrich-Heine University Hospital Dusseldorf (Director: Univ. Prof. Dr. med. R. Krauspe), Moorenstr. 5, D-40225 Dusseldorf, Germany*

**Keywords:** arthroplasty, BMP, bone defect, stem cells, total hip replacement

## Abstract

**Objective:**

Besides others, there are two major problems in total hip replacement surgery which result in implant failure. First there is aseptic loosening due to a lack of implant biocompatibility or micromovements and second periimplant wear debris induced osteolysis which limits the survival rate of an implant. Regarding to recent data there are only limited therapeutic strategies to heal these bony defects without arthroplasty revision surgery. Since the investigation and characterization of adult mesenchymal stem cells (MSCs) from bone marrow, a cell and tissue engineering based therapy might be a promising solution to heal endoprosthesis associated bony defects. Moreover the application of growth factors in bone reconstructive surgery is another treatment concept to promote local bone regeneration.

**Patient and Methods:**

We report about a 73-year old patient with a painful weight bearing and a large, wear debris induced pelvic osteolysis after total hip arthroplasty. To prevent from salvage surgical procedures and preserve bone, a healing attempted was performed by filling the critical bony defect zone with a BMP-2/MSC composit.

**Results:**

Clinical and radiological follow-ups showed a progressive bony healing of the critical size defect area without any complications. Fifteen months after application the patient is still pain free, has no limitations in daily life or sport activities.

**Conclusion:**

The case embarks on a strategy of non-embryonic stem cell and growth factor application to heal bony defects at patients with total hip endoprosthesis.

## INTRODUCTION

Up to date total hip replacement is the golden standard for the treatment of painfull osteoarthritis of the hip in advanced stages. Although the average survival rate of most implants is more than 10 years, the increase of the average lifetime of the population will make revision surgery to a bigger problem in near future.

Besides low grade infections and septic loosenings, the debris and cement disease are major reasons for an early aseptic implant loosening after total hip replacement (14). Depending on the particle size and chemical properties, polyethylene particles and metallosis can induce foreign body reactions which may induce local inflammation and peri-prosthetic osteolysis. As a biomechanical consequence, these local bone defects conduct progressive implant micromovements and may lead to endoprosthesis failure. Consequently various grades of critical bone defects (CSD) are more often noted in total hip revision surgery. Moreover the alternatives to reconstruct bony defects caused by wear debris are limited.

The use of allo- or autogenic bone grafts, the application of bone substitutes (biomaterials), osteosynthesis implants, or large revision arthroplasties are characterized by a lack of biological activity for bone regeneration and/or include biomechanical disadvantages. Therefore these techniques may lead to poor results in several cases. Especially the reconstruction of large bony defects at the pelvic site of a cup implant is technical demanding and bares a high risik of complications. Allogene transplantation of bone for the treatment of bony defects requires complex logistics affords. Besides high costs it is associated with several problems including infection risks, whereas autologous bone marrow transplantation is characterized by a donor site morbidity and limited resources. Furthermore it was shown in the literature that allograft bone transplants decreases in strength and weakens the periimplant region *in vivo* over time (36). As demonstrated by Kessler *et al*., 2003 (17) specially the duration of the revision surgery appears as a predictive parameter for perioperative morbidity in revision total hip arthroplasty.

One promising option in the treatment of osseous defects in endoprothesis revision surgery may be the local application of bone marrow derived mesenchymal stem cells (MSCs). It was shown by several investigators that the human bone marrow accomodates multipotent progenitor cells, which can differentiate into osteoblasts, chondroblast, myoblasts and adipoblasts (3, 4, 11, 12, 22, 30). Other authors demonstrated that the number of bone marrow derived MSCs can be significantly increased by vacuum aspiration techniques (26-28).

Although it has been shown in several pre-clinical studies that MSCs are potent to regenerate osteoblasts *in vitro* and *in vivo* (1) additional osteogenic stimuli may increase the osteoblastic potency of these cells. However, it is unclear if this strategy is helpful to heal large periimplant osteolytic defects.

An option to enhance the recruitment and promote the differentiation of osteoblasts in progenitor cells is the application of growth factors. One promising group of growth factors are the multi-functional bone morphogenetic proteins (BMPs). Belonging to the transforming growth factor beta (TGF-β) superfamily, the roles of BMPs in embryonic development and for cellular functions in postnatal and adult animals have been extensively studied in recent years. BMP signaling plays a crucial role in heart, neural and cartilage development at different molecular levels. Moreover it is shown that the BMP-2 subtype is one of the most promising bone promoting growth factors, which was already applied to heal local bone defects in pre-clinical and clinical trials (31).

In orthopedic and dental surgery BMP-2 was successfully applied to heal open tibial fractures, promote spinal fusions and augmentate or preserve the alveolar bone in the dental ridge (10, 19, 34).

Although BMP-2 can accelerate or improve bone healing when used alone, long healing times and abnormalities, such as cystic areas or abnormal microarchitecture of the new formed bone still occur (20, 32). Therefore there is a rational for an interest to combine BMPs with MSCs to heal bony defects.

Up to date there are only few clinical and experimental data about the application of BMPs in pelvis or hip. We report on a 73-year old male patient with severe debris induced acetabular osteolysis who has been treated by a BMP-2/MSC composit.

## PATIENT AND METHODS

A 73-year old male patient with progressive pain in the left groin and gluteal region, which occurred since January 2004 was transmitted to our department.

The patient underwent total hip replacement eight years ago (54 mm in diameter cementless threaded cup type “Münchner Schraubring” combined with a cementless stem type “Bicontact”/Merck, Germany and a ceramic head size medium).

**Figure 1:**
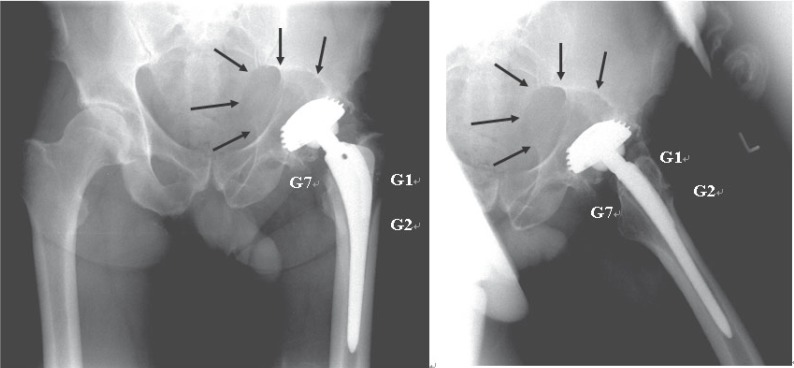
The X-rays of the pelvis in anterior-posterior (a) and axial planes (b) showed a thread cup with a large, expansive critical bony defect corresponding to a wear induced osteolysis. Moreover there are slight radiolucent lines at the proximal femur in Gruen-zones 1 (G1), 2 (G2) and 7 (G7).

The x-rays of the pelvis in two standard planes showed a condition after total hip arthroplasty with a large osteolytic tumor medial to the cup (7.5 × 4.0 × 3.5 cm in diameter, corresponding to 90 cm^3^). Furthermore there were only slight signs of a radiolucent line at the proximal femur corresponding to Gruen zones 1, 2 and 7 (13) without any additional signs of aseptic loosening (Figure 1).

Further diagnoses were a paroxysmal atrial fibrillation, arterial hypertension and a prostata carcinoma, which was treated by a transurethral protatectomy five years ago.

**Figure 2:**
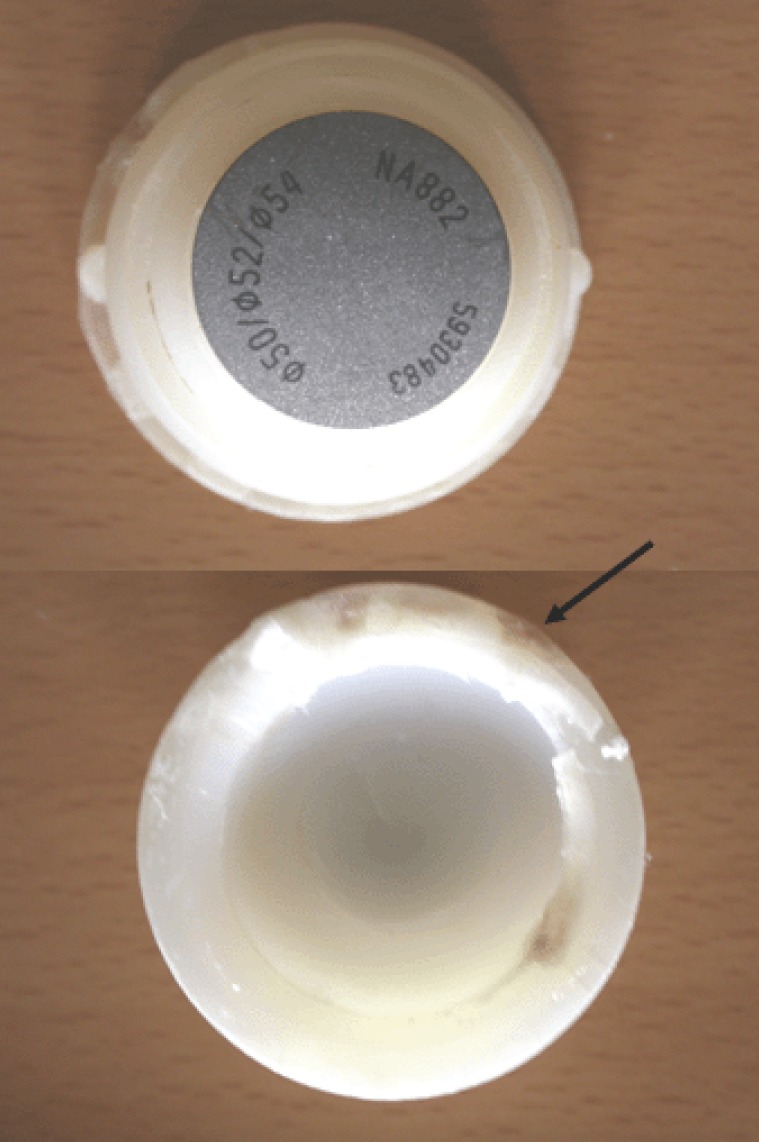
The liner of explanted thread cup showed a decentralized polyethylene wear (arrrow). Corresponding to a liner-neck impingement of the implant we found a brown-grey change in color on the polyethylene rim. Because the metal cup was designed with a central gap polyethylene particles could migrate behind the implant and cause wear debris inducing osteolysis at the inner pelvic site.

To exclude a malignant tumor we took a biopsy of the osteolytic pelvic area performing an anterior surgical approach to the pelvis. The histopathological representative tissue scanned under polarization microscopy showed a birefringent material corresponding to polyethylene wear debris. The laboratory blood parameters were uneventfull. In a second operation we replaced the worn out polyethylene liner (Figure 2). At this time the intraoperative examination of the prosthesis showed no sings of implant instability. To avoid a total cup replacement a healing attempt with a BMP-2/MSC composit was recommended to the patient. In accordance to the Declaration of Helsinki in its present form we informed the patient about BMP-2 associated perioperative risks including ectopic bone formation, allergic reactions and the formation of antibodies against BMP-2. The patient agreed with underwritten, informed consent.

Eight days later the osteolytic pelvic area was curettaged in a third stage surgery. Afterwards a total volume of 20 ml bone marrow aspirate at the posterior ilium was taken by Jamshidi-vacuum aspiration. Furthermore autologous spongiosa was obtained from the anterior iliac crest. Two collagen I/III sponges were sprinkled with 24 mg BMP-2 (Induct Os™/Sofamor).

To guarantee a sufficient protein adherence on the collagen surface we incubated the sponges for 15 min. at room temperature.

**Figure 3:**
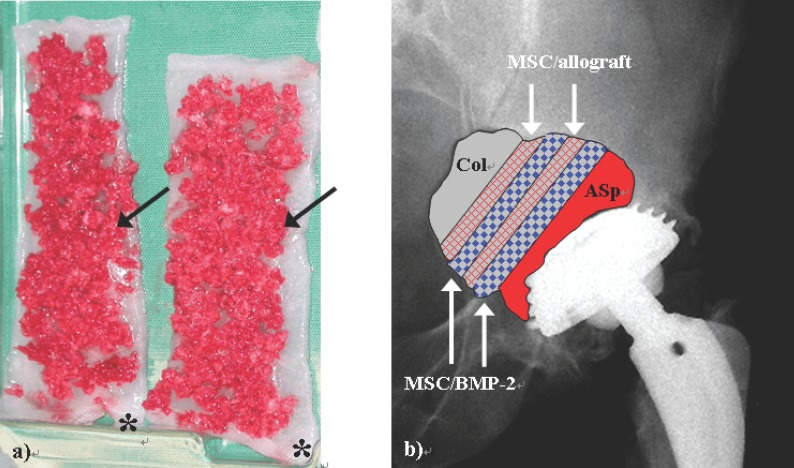
a) The figure shows two collagen I/III strips (*) which were impregnated by BMP-2, covered by MSCs containing autologous human bone marrow which was harvested by vacuum aspiration and augmented by spongiosa chips from iliac crest (arrows). b) The x-ray on a.p. view shows the multilayer sandwich-technique of refilling the critical bony osteolysis behind the cup. To lower the risk of an ectopic bone formation at the inner pelvic site collagen I/III sponges should act as a barrier and were positioned at the medical site of the reconstructive area (Col). Allograft bone was augmented by MSC containing bone marrow aspirate (MSC/allograft) and BMP-2 containing collagen I/III sponges were incubated with MSCs (MSC/BMP-2). Col: collagen I/III sponge, MSC(s): bone marrow derived mesenchymal stem cell(s), ASp: autologous spongiosa, BMP: bone morphogenic protein.

Afterwards the bone marrow cell suspension was distributed over the collagen I/III carrier and incubated for another 5 min. The bony defect cavity was filled in a multilayer sandwich-technique by collagen sponges, BMP-2/MSC-composits and allogen and autogen spongious transplants as shown in Figure 3. The total volume of the autologous bone transplant was less than one third of the whole pelvic defect.

## RESULTS

**Figure 4:**
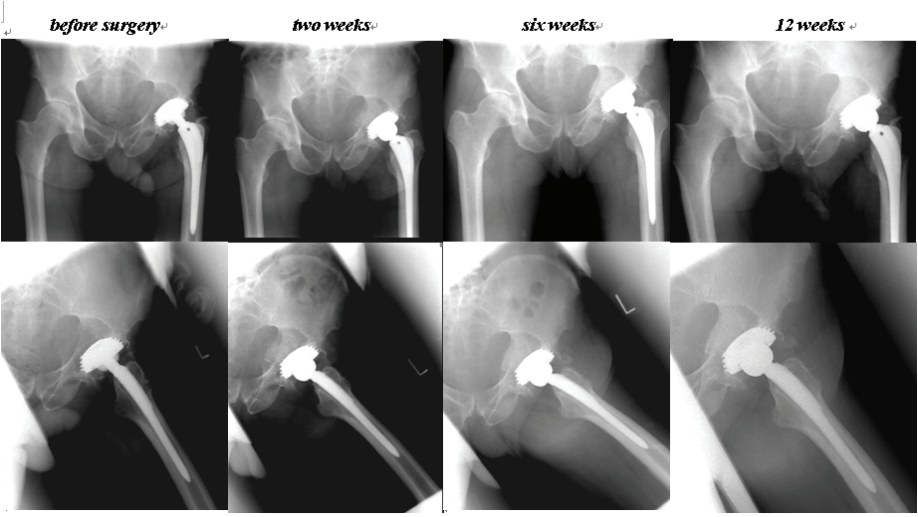
The figure shows x-rays in two standard planes of pelvis/hip. The roentgenological follow-ups before surgery and after two, six and 12 weeks after BMP-2/MSC-tretament show a progressive bony healing of the osteolytic area behind the cup.

The postoperative phase was uneventfull without any perioperative complication. The patient was mobilized on crutches with limited weight bearing of 20 kg at the left lower extremity for the first 12 weeks. After initial wound healing the patient was free of pain within 7 days. The patient was discharged after three weeks postoperatively and underwent a further physiotherapy program. X-ray controls of the pelvis in two standard planes after two, six, and 12 weeks showed a progressive osseous healing with newly formed bone in the osteolytic area (Figure 4). There were no immunoreactive antibodies against BMP-2 shown by serum immunophoresis after 12 weeks.

**Figure 5:**
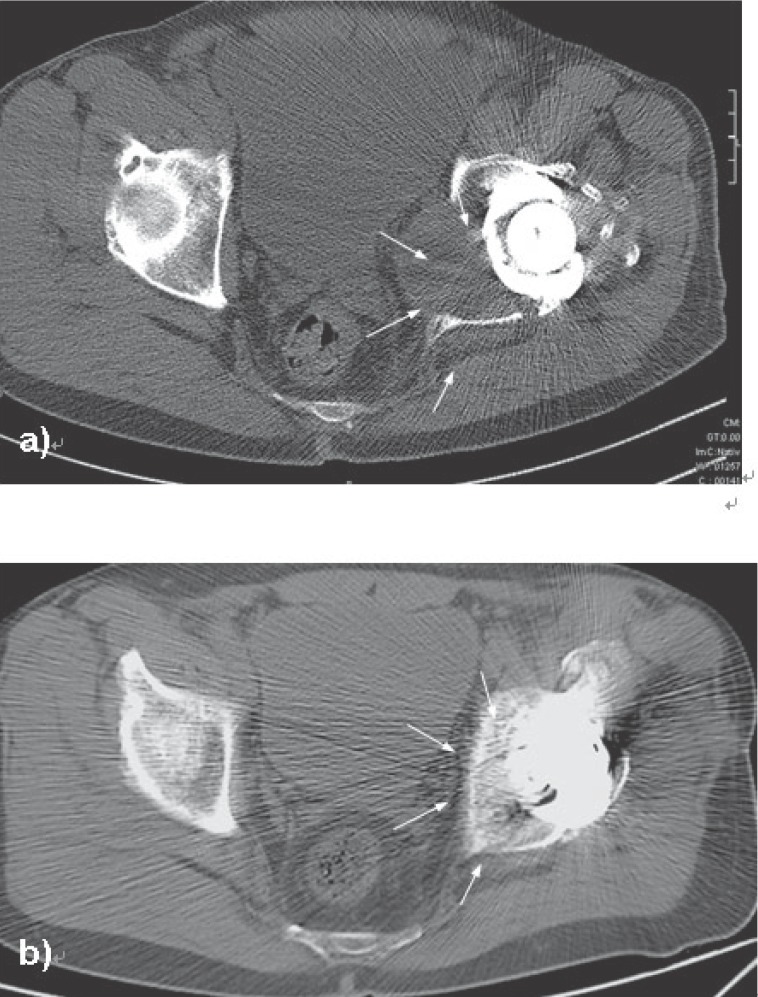
CT-scan before (a) and six months after (b) implantation of a BMP2/MSC-composit which was augmented by autologous and allogen spongious bone into an osteolytic pelvic cavity. a) The wear debris induced pelvic osteolysis lead to a pseudo-tumour with inflammative soft tissue. The inner cortical wall behind the cup is completely destructed whereas a cortical ring allows still a sufficient fixation of the thread cup. As a result to foreign body reaction ipsilateral pelvic organs are shifted by inflammatory tissue. The border of the expanding pseudotumour is marked by arrows. b) Six months after cuerettage of the pseudo-tumour and implantation of BMP-2/MSC-composit the initial critical size osteolysis is replaced by bone.

Six months after BMP-2/MSC application CT scans proof that the osteolytic cavity was completely filled out with solid bone (Figure 5). At this time and at latest follow up (fifteen months after surgery) the patient was free of pain and the range of motion (ROM) was unchanged compared to the preoperative status (flexion/extension: 110°/0°/10°, external/internal rotation: 40°/0°/30°, abduction/adduction: 30°/0°/10°). Three months after surgery the patient returned to sport activities (biking and hiking) and had no limitations in walking distances or daily life activities.

As far as we know this is the first successfull BMP-2/MSC application in wear induced osteolysis of the pelvis.

## DISCUSSIONS

In this study we showed that a local BMP-2/MSC application can promote bone formation within a wear debris induced peri-prosthetic osteolytic area. Our result corresponds to data of other authors in literature. Mont *et al*., 2003 (24) applied BMP-enriched allografts in osteonecrosis of the femoral head to avoid donor site morbidity in 19 patients. They implanted a combination of demineralized bone matrix (DBM), processed allograft bone chips, and a thermoplastic carrier through a window at the femoral head-neck junction. Mont *et al*., 2003 (24) presented a clinical success in 86% of the cases after a mean follow-up of 48 months. The positive influence of MSCs for bone regeneration is confirmed by Muschler *et al*., 2005 (29) who showed in a canine spinal fusion model that a MSC-enriched matrix graft has advantages in bone healing compared to a matrix graft which is augmented by bone marrow.

In another study Bragdon *et al*., 2003 (2) applied BMP-2 in a canine total hip arthroplasty model at the porous acetabular component and demonstrated a local promotion of the defect filling and bone ingrowth into the porous coating beneath the defect region by rhBMP-2/calcium phosphate cement (α-BSM^®^).

Murakami *et al*., 2003 (25) investigated the rhBMP-2 related bone formation using poly-D,L-lactic-acid-para-dioxanone-polyethyleneglycol block co-polymer (PLA-DX-PEG) carrier in a canine model. To induce a critical femoral defect, half of the proximal femur was surgically resected and treated by the BMP-2/PLA-DX-PEG composit. They showed a bone repair after a 12 weeks follow-up and advocate the use of BMP-2-“hybrid” prosthesis as a new modality to repair bone defects in endoprosthesis revision surgery.

In contrast to the promising experimental and clinical data mentioned before some authors report about negative results after BMP application. McGee *et al*., 2004 (23) investigated in their pilot study the effect of BMP-7 on bone graft incorporation in sheep´ femurs impacted with allograft after cemented hemiarthroplasty. They found an increased initial graft resorption and associated stem subsidence in the BMP-7 group. McGee *et al*., 2004 (23) pointed out the demand of further studies examining dose response effects. They advised against clinical application of BMP-7 in femoral impaction grafting at revision hip arthroplasty.

In contrast to McGee *et al*., 2004 (23) we used a BMP-2/MSC composit to promote the local healing in wear debris induce osteolysis which was augmented by allograft an autologous spongiosa.

Although it is demonstrated in several studies that BMP-2 promotes an ectopic bone formation in animal MSCs, there is still a controversial discussion about the effects of BMP-2 on human MSCs in literature.

Diefenderfer *et al*., 2003 (8) investigated the BMP signaling pathways in human bone marrow derived MSCs. In their study with MSC samples from more than a dozen patients, only one patient sample showed a significantly elevated alkaline phosphatase (ALP) after exposure to BMP whereas the rest responded to dexamethasone but not to BMP. As a consequence Diefenderfer *et al*., 2003 (7) concluded that dexamethasone is required for induction of the osteoblast phenotype. The importance of dexamethasone presence and its necessity in osteoblast differentiation pathways (7) correspond to the data of our group published previously (15).

Based on trauma related stress it is evident that the glucocorticoid levels are increased in patients after surgery. As demonstrated by Wellby *et al*., 1994 (35) it is evident that the cortisol concentration increases after 30 minutes and peaked at four hours after elective surgeries.

In conclusion the *in vivo* bone formation promoted by BMP-2/MSC composit may not only base on BMP-2/MSCs but also influenced by surgery related increased level of glucocorticoides. Furthermore sex and age of a patient may also influence BMP/MSC associated bone healing. Muschler *et al*. 1997, 2001, 2002 (26-28) reported a significant age-related decline in the number of nucleated cells harvested per bone marrow aspirate for both men and women. However, they demonstrated that the number of colony forming units (CFU) which express alkaline phosphatase decreased significantly with age for women, but not for men.

Diefenderfer *et al*., 2003 (7) showed advantages of BMP-2 for the differentiation of osteoblasts in MSCs compared to BMP-4 and -7. They presented a significantly higher induction of ALP in males.

Another factor which may influence the osteoinductive effects of BMP-2 and MSCs *in vivo* is the applied scaffold. In this case report we used a collagen I/III carrier as a BMP-2 and MSC scaffold. The finding that collagen I/III as a suitable carrier for MSCs with a low degree of cytotoxicity and osteoinductive properties is confirmed by our *in vitro*-studies published previously (14-16, 37). The qualification of three dimensional collagen I/III as an appropriate scaffold for MSCs based bone and cartilage engineering was also confirmed by (38) and Liu (21). It is evident that especially collagen I, a normal constituent of bone, plays an important role in osteoblast differentiation and function. Kihara *et al*. (18) showed on rat MSCs that osteoblastic differentiation is enhanced by adding solubilized type I collagen. Moreover Farrel *et al*., 2006 (9) demonstrated that a collagen-I-glycosaminoglycan composit allows an osteogenic differentiation of rat MSCs *in vitro*. These data are coressponding to Chastain *et al*., 2006 (5) who showed that collagen I acts as a mediator for MSCs to attach to polylactide scaffolds.

A promising alternative to BMP-2 application to enhance the osteogenic potency of MSCs may the use of BMP-2 transfected stem cells. Tsuda *et al*., 2005 (33) reported about an enhanced osteoinduction by MSCs transfected with a fiber-mutant adenoviral BMP-2 gene in rats. In another study Dai *et al*., 2005 (6) demonstrated on a goat diaphyseal defect that a combination of biphasic calcined bone (BCB) and MSCs transduced with hBMP-2 significantly improved the bone healing. However, it is unclear if gene therapeutic strategies will fulfil the demands of biological safety and efficiency under clinical application.

Although this case showed a rapid healing of the critical size osseous defect, there is no evidence that BMP-2 application was solely responsible for healing of the severe osteolysis.

However, there are some arguments that BMP-2 supports the bone healing significantly in our case: The patient was an elderly men and not a young person with high bone healing potential 1). The defect zone was critical in size for a spontaneous healing 2) and the total volume of the autologous bone graft was less than one third of the whole defect 3). The presence of wear debris and inflammative soft tissue has a negative influence on bone healing 4), but the patient showed a rapid bone healing within 12 weeks after the MSC/BMP-2 transplantation 5).

It is not clear if the patient would have benefit from a two-stage surgical procedure (1. biopsy, 2. implant exchange and defect filling) which would reduce costs and hospital stay. Overall, individual factors (co-morbidity, size of defect, quality of bone, surgical opportunities and experiences, demands of the patient for sports and daily activities) has to be considered in decision making for or against an BMP-2 application in the treatment of patients with a periimplant osseous defect. In contrast to a conventional one-stage revision surgery including an implant exchange and defect filling by bone substitutes (autologous/allogene bone grafs, hydroxyapatite, tricalciumphosphate) a two or three-stage procedure has the disadvantage of repititive anaesthesias and prolonged hospital stay.

## CONCLUSIONS

Considering individual facts the local application of BMP-2/MSC may restore lost bone mass encountered in revision arthroplasty.
